# Tuberculosis Topics

**Published:** 1917-10-27

**Authors:** 


### TUBERCULOSIS TOPICS.

### The Welsh National Memorial Association.

In peace-time an annual report appearing in the autumn would be unseasonable, but during a European war it must be reckoned inevitable. The Welsh National Memorial Association has issued a neat brochure recording its activities for the fifth year of its existence. The well-known name of Mr. Gwilym Hughes, its secretary, appears this year in connection, unfortunately, with a resignation owing to ill-health. The growth in the work of the association is strikingly shown under the heading "Provision of Institutions," from which it appears that "previous to July 1, 1912, there were in existence only thirty-one beds in Wales and Monmouthshire in institutions dealing exclusively with the treatment of tuberculosis; that number in less than five years has been increased to more than a thousand." In view of the present war, with its crop of tuberculous combatants, that result is most happy and timely. The demand for beds is stated to be as great as ever, and particularly beds for patients suffering with surgical tuberculosis. On the financial side, we learn from the address of the President, Major Davies, M.P., that the necessary contribution of the authorities to the excess expenditure is likely to be nearly four times what it was in the preceding year. The main causes for this are the diminished income from Insurance Committees, and the great increase in the cost of commodities. The Treasury has already intimated its willingness to bear its proportion of the excess expenditure, and the local authorities—the County and Borough Councils of Wales—have agreed to withdraw the limit of a halfpenny rate, substituting one of three-farthings.

### A Chair of Tuberculosis.

Tuberculosis specialists all over the country will be gratified at the now realised plan of Edinburgh University to institute a Chair of Tuberculosis and to appoint thereto Sir Robert Philip. For this disease, the commonest of any magnitude affecting, at any rate, the white races, has become a subject the scope of which may take the whole time and powers of any man. Indeed, it would be a fitting action were the new professor now to resign his other medical appointments; it would be an example in concentration the reverberations of which would greatly tend to increase the national medical efficiency. The general physician, no matter how great his administrative zeal and ability, can no longer compete as a phthisiologist with, say, the Swiss clinicians, just because the time and energy and opportunity at their disposal are necessarily greater than his own.

